# Supplementary material for: G Protein-Coupled Receptor 17 Inhibits Glucagon-like Peptide-1 Secretion via a Gi/o-Dependent Mechanism in Enteroendocrine Cells
Source: Biomolecules. 2024 Dec 25;15(1):9. doi: 10.3390/biom15010009 (PMC11762167; doi:10.3390/biom15010009)
Supplement: Supplementary file 1 [file biomolecules-15-00009-s001.zip › 2024-11-06_Conley, et al. Biomolecules_Supplementary Materials.pdf]

Supplementary Figure 1

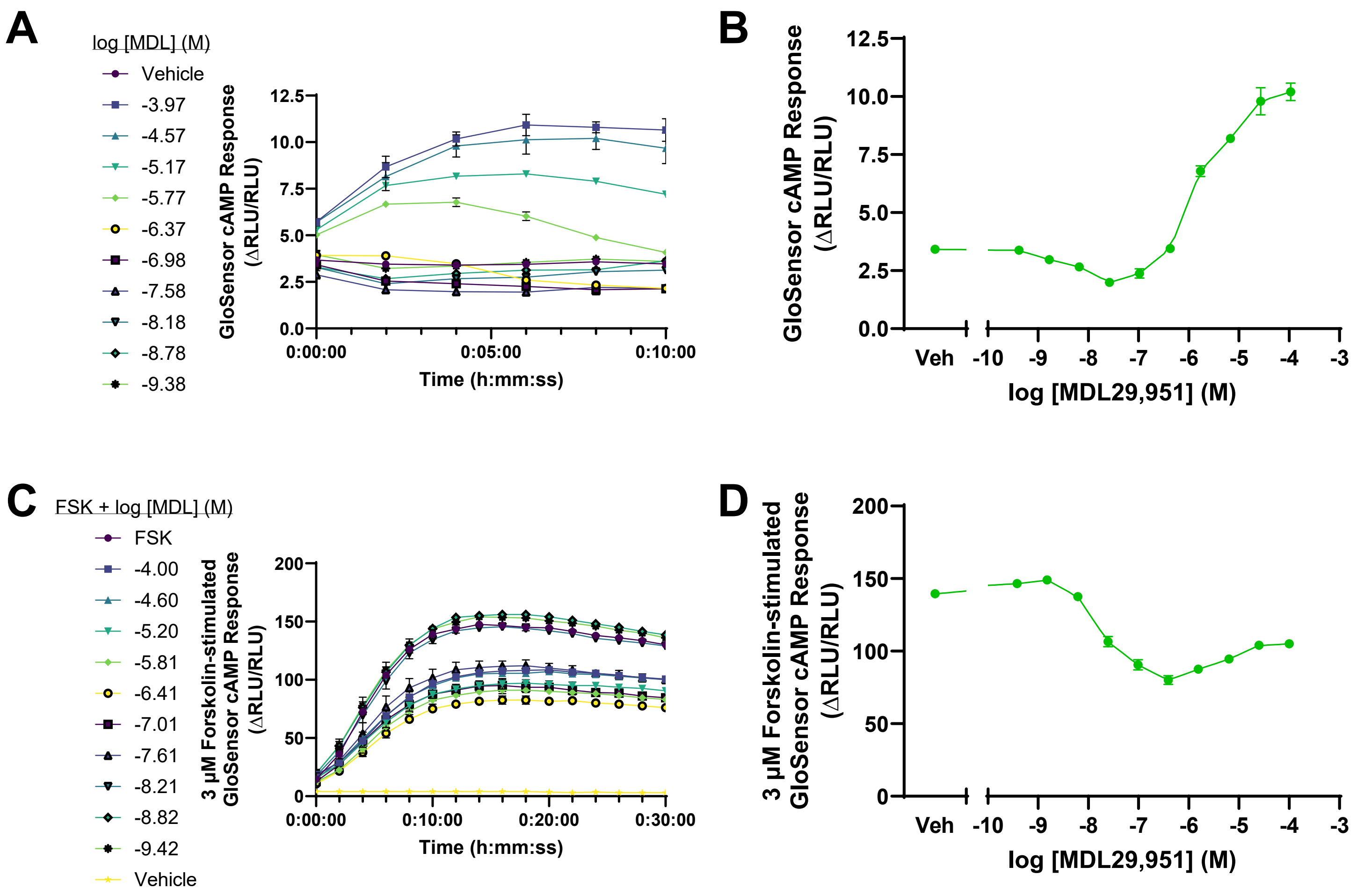

Figure S1. Human GPR17 long isoform modulates cAMP in GLUTag cells. (A) GloSensor cAMP luminescence responses were measured in GLUTag cells transiently transfected with hGPR17L and pGloSensor-cAMP-22F. Cells were treated with 500 µM IBMX and then stimulated with indicated concentrations of MDL29,951 and the luminescence response was measured every two minutes for ten minutes. **(B)** Concentration-response curve for MDL29,951 effects on GloSensor cAMP response four minutes after treatment. **(C)** GloSensor cAMP luminescence responses were subsequently measured in the same cells after stimulation with 3 µM forskolin. **(D)** Concentration-response curve for MDL29,951 effects on GloSensor cAMP signal following treatment with 3 µM forskolin. Data points represent the average responses for measurements between 16 and 30 minutes after forskolin stimulation. Data represent mean±SEM of two independent experiments performed in duplicate.

Supplementary Figure 2

A

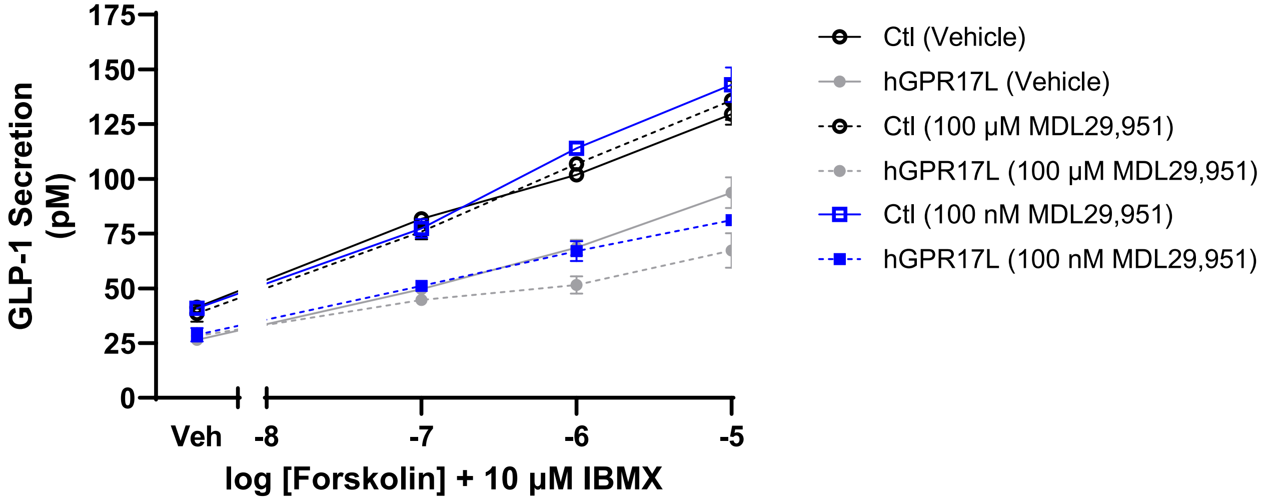

B

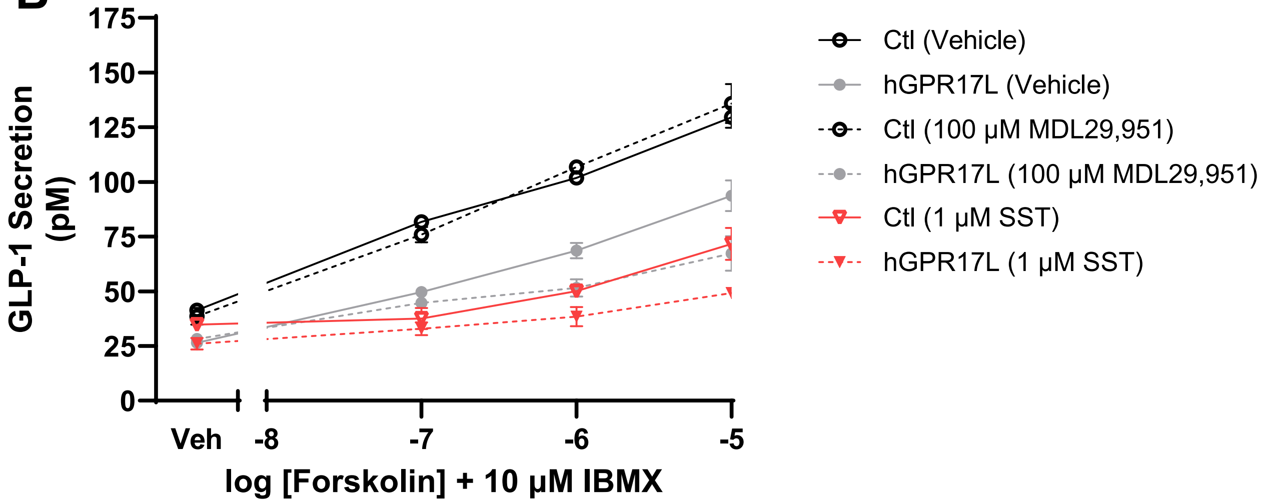

Figure S2. Somatostatin negatively regulates GLP-1 secretion from GLUTag cells. GLP-1 secretion was measured from control or hGPR17L-expressing GLUTag cells that were treated with **(A)** 100 nM MDL29,951 or **(B)** 1 μM somatostatin (SST) together with varying concentrations of forskolin and 10 μM IBMX. GLP-1 secretion in response to vehicle or 100 μM MDL29,951 treatments from Figure 3C are shown for reference comparison. Data are mean ± SEM of two independent experiments.

Supplementary Figure 3 and Table S1

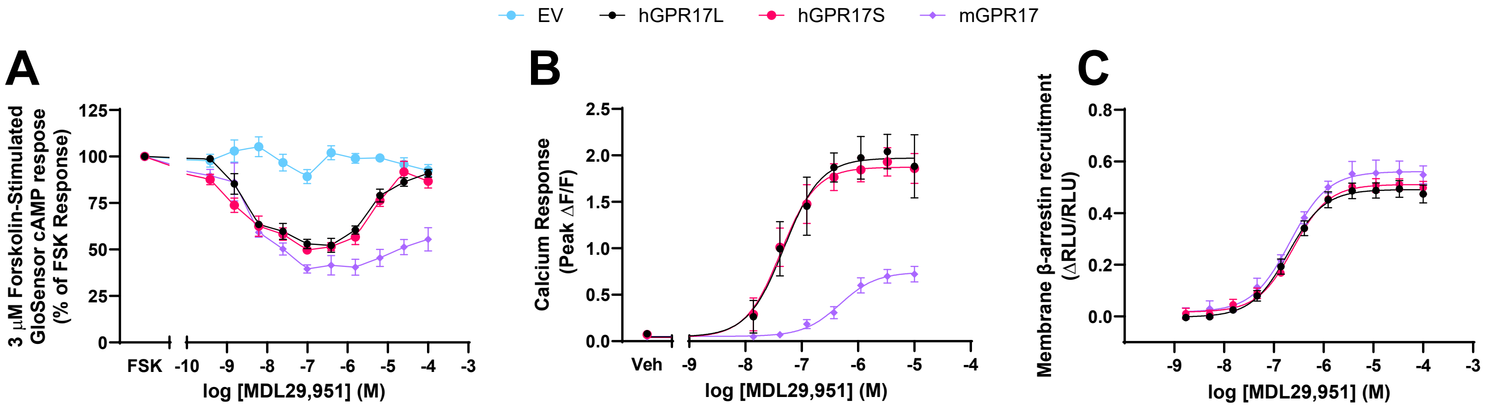

Figure S3. Human GPR17 and mGPR17 have distinct second messenger signaling profiles in HEK293 cells. HEK293 cells were transiently transfected with hGPR17L, hGPR17S, mGPR17, or empty vector (EV) and second messenger signaling regulation was measured. Concentration-response curves for MDL29,951 effects on **(A)** 3 μM forskolin-stimulated GloSensor cAMP signal, **(B)** calcium mobilization, and **(C)** membrane β-arrestin recruitment. Data represent mean±SEM of three to four independent experiments.

Table S1. Human GPR17 and mGPR17 have distinct second messenger signaling profiles in HEK293 cells. Data represent summaries of the potency and efficacy measures for concentration-response analyses of MDL29,951 effects on 3 μM forskolin-stimulated GloSensor cAMP signal, calcium mobilization, and membrane β-arrestin recruitment that were reported in Figure S3A, S3B, and S3C, respectively. Data represent mean±SEM of three to four independent experiments. Efficacy measures were statistically analyzed by one-way ANOVA with Dunnett's multiple comparisons test as compared to the hGPR17L transfection condition. * $p < 0.05$, ** $p < 0.01$, *** $p < 0.001$. EV, empty vector. nd, not determined.

	cAMP				Calcium		β-arrestin	
Receptor	Potency IC$_{50}$ (nM) (pIC50)	Maximum Inhibition (% Inhibition)	Potency EC$_{50}$ (μM) (pEC50)	Effect of 100 μM MDL (% Inhibition)	Potency EC$_{50}$ (nM) (pEC50)	Emax (Mean Peak ΔF/F)	Potency EC$_{50}$ (nM) (pEC50)	Emax (Peak ΔRLU/RLU)
hGPR17L	3.0 (8.52±0.08)	48±2.9	4.0 (5.40±0.09)	9.0±2.1	51 (7.29±0.11)	1.92±0.22	190 (6.72±0.09)	0.50±0.03
hGPR17S	1.5 (8.83±0.09)	48±2.2	3.6 (5.45±0.07)	13±3.7	43 (7.37±0.10)	1.84±0.11	240 (6.62±0.08)	0.50±0.04
mGPR17	3.3 (8.48±0.20)	61±3.4*	18 (4.74±0.23)	45±6.3***	490 (6.31±0.05)	0.70±0.09**	200 (6.69±0.10)	0.54±0.03
EV	nd	nd	nd	7.5±3.2	nd	nd	nd	nd

Supplementary Figure 4

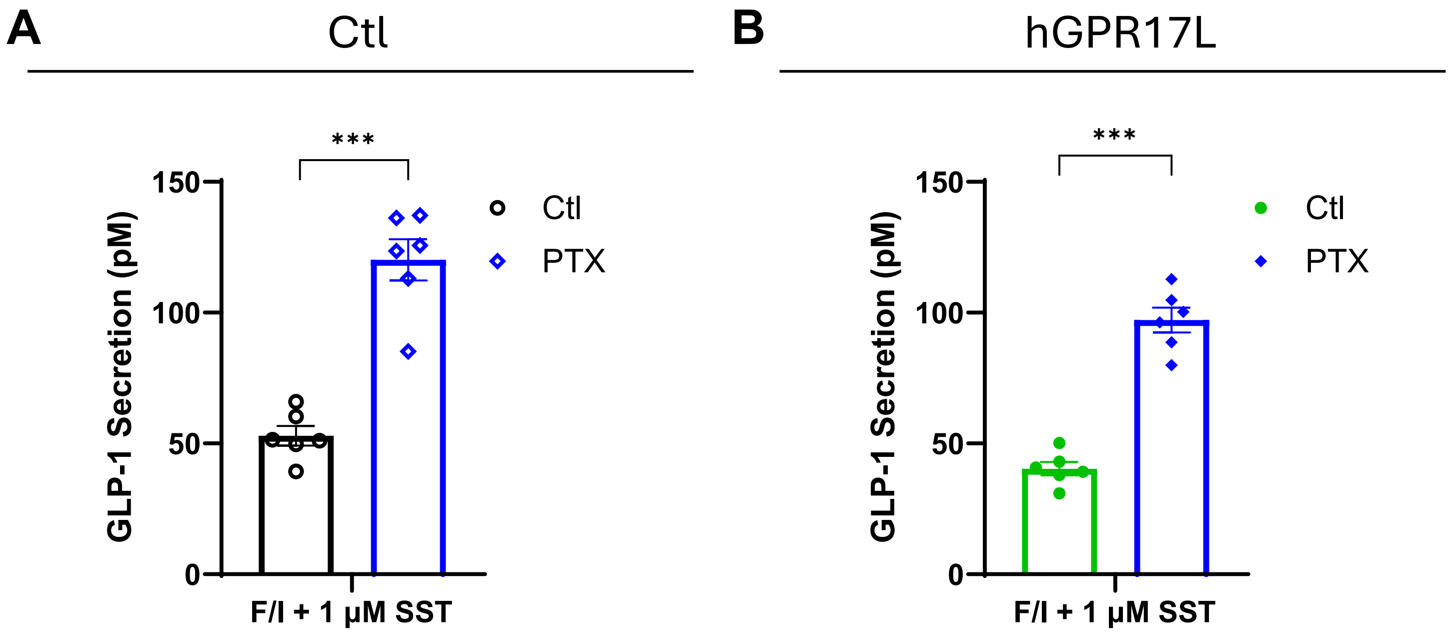

C

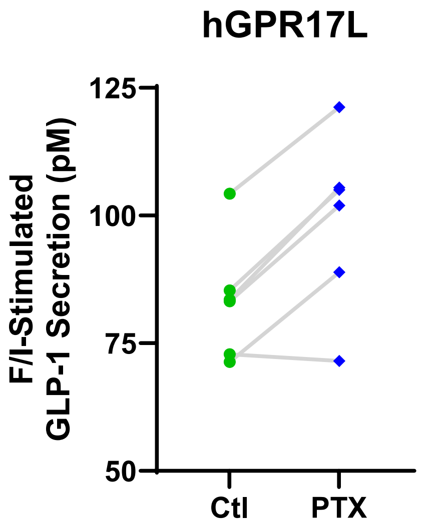

Transduction	hGPR17L	hGPR17L	
Treatment 1	Ctl	PTX	
Treatment 2	Vehicle	Vehicle	
Treatment 3	10 µM F/I	10 µM F/I	
	GLP-1 Secretion (pM)		Δ
Rep1	104.3	121.2	17.0
Rep2	83.2	102.0	18.8
Rep3	72.9	71.6	-1.3
Rep4	83.5	105.5	22.0
Rep5	71.3	88.9	17.6
Rep6	85.3	105.0	19.7
Mean	**83.4**	**99.0**	**15.6**
SEM	4.8	6.9	3.5

Figure S4. Pertussis toxin attenuates somatostatin-mediated inhibition of GLP-1 secretion and hGPR17L-mediated constitutive inhibition of GLP-1 secretion. GLUTag cells were transduced with **(A)** control adenovirus or **(B)** adenovirus encoding hGPR17L, and GLP-1 secretion was measured in cells that were treated as indicated together with either control or 100 ng/mL PTX. Data represent mean ± SEM for five or six independent experiments and were analyzed with unpaired t tests comparing matched control and PTX treated conditions. ***, $p < 0.001$. **(C)** Panel and table of data from Figure 5B demonstrating effects of PTX on F/I-stimulated GLP-1 secretion from matched experiments in GLUTag cells expressing hGPR17L.

Supplementary Figure 5

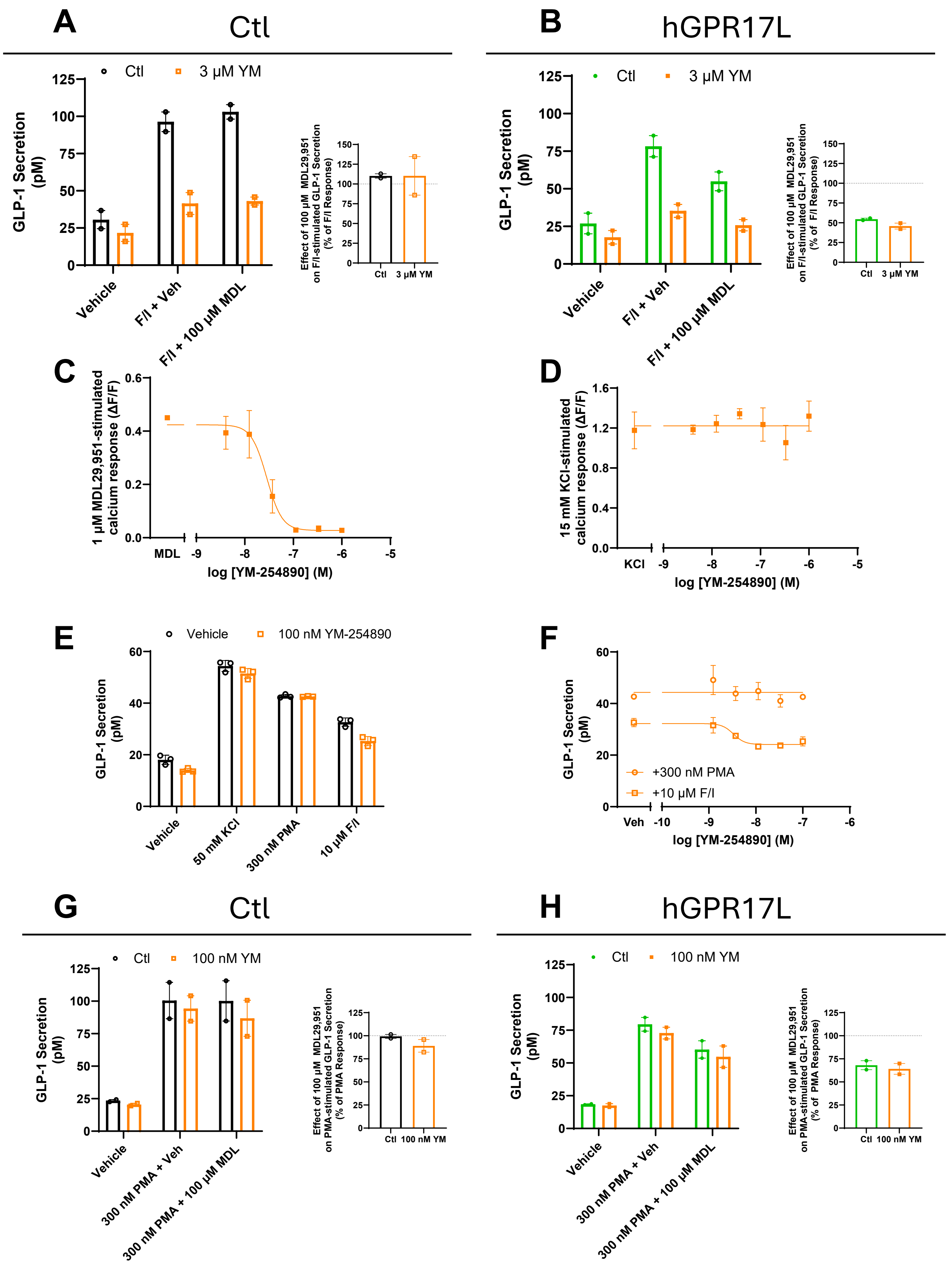

Figure S5. YM-254890 selectively inhibits GPR17 agonist-mediated calcium flux but has no effect on hGPR17L-mediated inhibition of GLP-1 secretion. GLUTag cells were transduced with **(A)** control adenovirus or **(B)** adenovirus encoding hGPR17L and GLP-1 secretion was measured in cells that were treated as indicated together with either control or 3 μM YM-254890. Data represent mean±SEM for two independent experiments performed in duplicate. GLUTag cells expressing hGPR17L were treated with indicated concentrations of YM-254890 and stimulated with **(C)** 1 μM MDL29,951 or **(D)** 15 mM KCl and calcium mobilization was measured. Data represent mean±SD of a single experiment performed in triplicate. GLP-1 secretion was measured from GLUTag cells that were stimulated as indicated in the presence of **(E)** control or 100 nM YM-254890 treatment or **(F)** indicated concentrations of YM-254890. Data represent mean±SD of a single experiment performed in triplicate. GLUTag cells were transduced with **(G)** control adenovirus or **(H)** adenovirus encoding hGPR17L and GLP-1 secretion was measured in cells that were treated as indicated together with either control or 100 nM YM-254890. Data represent mean±SEM for two independent experiments performed in duplicate.

Supplementary Figure 6

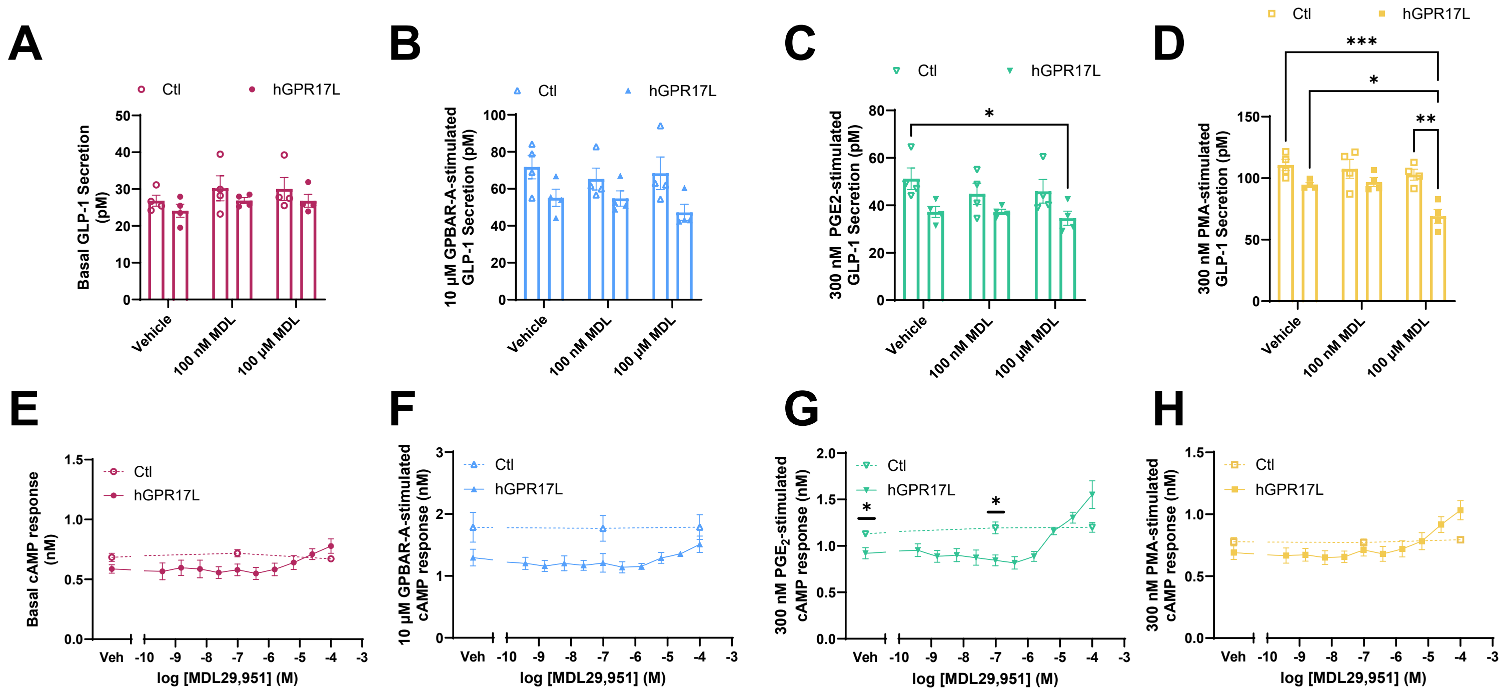

Figure S6. Human GPR17 long isoform distinctly regulates GLP-1 secretion and cAMP signaling in a manner that is dependent on co-stimulation conditions in GLUTag cells. GLUTag cells were transduced with control adenovirus or adenovirus encoding hGPR17L and GLP-1 secretion was measured in response to treatment with **(A)** vehicle, **(B)** 10 µM GPBAR-A, **(C)** 300 nM PGE_2, or **(D)** 300 nM PMA together with vehicle, 100 nM MDL29,951, or 100 µM MDL29,951. Data represent mean±SEM of four independent experiments performed in triplicate and were analyzed using one-way ANOVA with Sidak's post hoc test. *, $p < 0.05$, **, $p < 0.01$, ***, $p < 0.001$. Cyclic AMP was measured under **(E)** vehicle, **(F)** 10 µM GPBAR-A, **(G)** 300 nM PGE2, or **(H)** 300 nM PMA stimulation conditions in combination with the indicated concentrations of MDL29,951 in control or hGPR17L-expressing GLUTag cells. Data represent mean±SEM for two to four independent experiments performed in triplicate and were analyzed by unpaired t test comparing matching treatment conditions between control and hGPR17L-expressing cells. *, $p < 0.05$.
